# Supplementary material for: Behavioural issues in late life may be the precursor of dementia- A cross sectional evidence from memory clinic of AIIMS, India
Source: PLoS One. 2020 Jun 10;15(6):e0234514. doi: 10.1371/journal.pone.0234514 (PMC7286493; doi:10.1371/journal.pone.0234514)
Supplement: Attachment — Submitted filename: Response to Reviewers.docx [file pone.0234514.s003.docx]

**DEPARTMENT OF GERIATRIC MEDICINE**

**ALL INDIA INSTITUTE OF MEDICAL SCIENCES**

**ANSARI NAGAR, NEW DELHI - 110029**

April 30, 2020

To,

Editor-in-chief,

Plos One

Dear Professor,

Hereby attaching response to reviewers below

1. Written informed consent was taken from participants. Ethical statement has been added under Materials and methods in the manuscript
2. –
3. Results section has been described in detail as advised
4. Footnotes have been added
5. No, Apo-E analysis has not been done of the study subjects
6. The tertiary care centre caters to the population of Delhi as well as nearby cities and states. This statement has also been mentioned in Materials and methods in the manuscript
7. One figure showing the comorbidities between the groups has been added
8. Discussion has been modified

Thank you for your help
